# Molecular Diagnosis and Quantification of Bovine Abortion Caused by *Toxoplasma gondii* in the Tropical Climate of Brazil

**DOI:** 10.1155/vmi/4914633

**Published:** 2026-08-26

**Authors:** Marco Túlio Dos Santos Costa, Marlon Ribeiro, Caroline Argenta Pescador, Milena Lima Bernardes, Valéria Dutra, Tatiana Gabriela Paz Calvache, Luciano Nakazato

**Affiliations:** ^1^ Department of Microbiology and Molecular Biology, College of Veterinary Medicine, Federal University of Mato Grosso, Av. Fernando Corrêa da Costa, 2367 Boa Esperança, Cuiabá, Mato Grosso, Brazil, ufmt.br; ^2^ Department of Animal Pathology, College of Veterinary Medicine, Federal University of Mato Grosso, Av. Fernando Corrêa da Costa, 2367 Boa Esperança, Cuiabá, Mato Grosso, Brazil, ufmt.br

**Keywords:** abortion, cattle, qPCR, toxoplasma

## Abstract

*Toxoplasma gondii* is an obligate intracellular parasite, considered a zoonotic agent, and one of the main causes of foodborne diseases in humans. It is also responsible for infectious abortion and congenital defects in ruminants and human fetuses. While abortion in small ruminants due to *T. gondii* is well documented in the literature, bovine abortion is rare. In this article, we investigate the occurrence of *T. gondii*‐induced abortion in cattle, providing molecular and immunohistochemical evidence from Brazilian herds. A total of 156 bovine fetuses were submitted for necropsy, and tissue samples were collected. Histopathology, immunohistochemistry, and real‐time quantitative polymerase chain reaction (qPCR) were performed on these samples. Differential diagnosis was conducted for five bovine abortion diseases. Three samples (1.9%) showed lesions compatible with *T. gondii* on histopathology and tested positive by qPCR, with quantities of 476 copies/mg, 148 copies/mg, and 16 copies/mg, respectively. One positive sample was purified and prepared for sequencing, confirming identification as *T. gondii*. This study demonstrates that circulating *T. gondii* in Brazil is capable of causing abortion in cattle, highlighting the need for specific prophylactic measures against the pathogen and the importance of avoiding management practices that favor its establishment.

## 1. Introduction

*Toxoplasma gondii* (*T. gondii*) is an obligate intracellular parasite of the phylum Apicomplexa that typically infects warm‐blooded animals and is widely distributed worldwide, with a broad range of animal and human hosts [1, 2].

Toxoplasmosis is a zoonosis and one of the main causes of foodborne illness in humans. It is also responsible for infectious abortion in both ruminants and humans [3, 4]. The transmission routes of *T. gondii* include ingestion of food or water contaminated with oocysts shed in the feces of infected cats, consumption of raw or undercooked meat containing tissue cysts, and vertical transmission from mother to fetus during pregnancy.

Congenital toxoplasmosis occurs when a pregnant female becomes infected, allowing the parasite to cross the placenta and enter the fetal circulation. The infection can progress in various ways, including fetal resorption, stillbirth, and abortion, with abortion being more common in small ruminants when infection occurs during the third trimester of gestation [5–7].

*T. gondii* is divided into three main genetic lineages based on its virulence in mice: Type I, Type II, and Type III. Type I strains are highly virulent and often associated with severe disease, while Type III strains are moderately and slightly virulent, respectively, and are more commonly found in human and animal infections, including congenital toxoplasmosis. In small ruminants, *T. gondii* is among the primary causes of abortion [8]; however, in cattle, such cases are less frequent [9]. The Brazilian cattle herd (both beef and dairy) represents a significant economic activity, yet data on bovine abortion due to *T. gondii* are scarce. Nonetheless, serological evidence of *T. gondii* infection in herds exists [10].

Therefore, the objective of this article is to provide a comprehensive description of the first confirmed case of bovine abortion caused by *T. gondii*, including necropsy findings, histopathological and immunohistochemical results, quantitative polymerase chain reaction (qPCR) quantification, and DNA sequencing, in order to characterize this rare abortifacient agent in Brazil.

## 2. Materials and Methods

Bovine fetus samples were referred for necropsy to the Veterinary Hospital of the Federal University of Mato Grosso (HOVET‐UFMT) between 2012 and 2022. A total of 156 samples were selected from submissions during this 10‐year period. The inclusion criteria were based on the availability of complete clinical history, adequate tissue preservation for histopathological and molecular analyses, and cases where abortion was the primary clinical presentation. The fetuses originated from multiple herds across the states of Goiás, Mato Grosso, and Minas Gerais. The samples were collected from dairy and beef cattle herds managed under extensive and semi‐intensive systems. Epidemiological and clinical data, including animal breed, geographical location, and fetal age, were collected at the time of the service request. Fetal ages were estimated based on crown‐rump length measurements and morphological characteristics during necropsy.

Samples of the brain, heart, lung, liver, kidney, spleen, and thymus were fixed in 10% buffered formalin, sectioned into 4 μm slices, and stained with hematoxylin and eosin for histopathological evaluation.

The presence of *T. gondii* was investigated in immunohistochemical sections of the brain, heart, and liver. Tissue samples embedded in paraffin blocks were sectioned using a microtome to obtain 3 μm slices, which were then mounted on glass slides. Brain tissue from a paraffin block with a PCR‐confirmed diagnosis, and also known as positive in IHQ, was used as a positive control.

Mounted tissue sections were washed twice in xylene for 20 min each, followed by sequential washes in 100%, 96%, 80%, and 70% alcohol for 2 minutes each. The slides were then washed in distilled water for 1 minute. Endogenous peroxidase activity was blocked using 30% hydrogen peroxide for 20 min, followed by washes in phosphate‐buffered saline (PBS) with Tween (a nonionic aqueous solution) and distilled water for 5 minutes each.

Antigen retrieval was performed in a water bath with citrate buffer using a microwave at 90°C for 4 min, ensuring no buffer evaporation. 5% Bovine serum albumin (BSA) (Kit Elabscience) was applied for 30 min to prevent nonspecific binding. The slides were then washed in PBS with Tween and distilled water for 5 minutes each.

A goat‐derived lyophilized anti‐*T. gondii* polyclonal antibody (VMRD) was used as the primary antibody, diluted 1:1000 in PBS, and incubated for 12 h at 4°C and 2 h at 37°C in an oven. Slides were again washed in PBS with Tween and distilled water for 5 min each.

An anti‐goat polyperoxidase IgG secondary antibody (Kit Elabscience) was applied for 20 min at room temperature, followed by washes in PBS with Tween and distilled water for 5 min each.

Chromogenic detection was performed using DAB for 2 minutes, and slides were counterstained with hematoxylin for 10 s. Dehydration was conducted using 80%, 96%, and 100% alcohol for 2 minutes each. Finally, the slides were cleared in xylene (two washes of 5 minutes each) and mounted for observation under an optical microscope.

Fifty milligrams of tissue samples (brain, heart, lung, liver, kidney, spleen, and thymus) were subjected to total nucleic acid extraction using the MagMAX Total Nucleic Acid Isolation Kit, following the manufacturer’s protocol. Extracted DNA was resuspended in 70 μL of the elution buffer provided in the kit.

The samples were analyzed for *T. gondii* by real‐time PCR using the Applied Biosystems QuantStudio 5 Real‐Time PCR System (Thermo Fisher Scientific, USA) and the commercial PowerUp SYBR Green Master Mix (Thermo Fisher Scientific, USA). Specific primers TOXO1 (5′‐GGAACTGCATCCGTTCATGAG‐3′) and TOXO2 (5′‐TCTTTAAAGCGTTCGTGGTC‐3′) were used to amplify a 194‐bp fragment of the B1 region [11]. A standard curve was generated using five serial 1:10 dilutions of a plasmid (pGEM) containing the cloned amplicon, starting from 2.1 × 10^5^ to 2.1 × 10^1^ copies.

Detection of other abortigenic pathogens was performed on tissues for Bovine herpesvirus type 1 (BoHV‐1), bovine viral diarrhea virus (BVDV), *Brucella abortus*, *Leptospira* spp., and *Neospora caninum* (Table 1).

**TABLE 1:** Abortigenic agents previously tested.

Agent	Primer	Reference
BoHV‐1	5′ CAA CCG AGA CGG AAA GCT CC 3′ 5′ AGT GCA CGT ACA GCG GCT CG 3′	Claus [12]

BVDV	N.A.^∗^	VetMAX‐Gold BVDV PI Detection Kit

*Brucella abortus*	5′GACGAACGGAATTTTTCCAATCCC3′ 5′TGCCGATCACTTAAGGGCCTTCAT3′	Bricker and Halling [13]

*Leptospira spp.*	5′ ATCTCCGTTGCACTCTTTGC 3′ 5′ ACCATCATCATCATCGTCCA3 ′	Ahmed et al. [14]

*N. caninum*	5′TAC TAC TCC CTG TGA GTT G 3′ 5′ TCT CTT CCC TCA AAC GCT 3′	Buxton [15]

^∗^N/A stands for “not available,” as the kit does not supply the primer sequence.

A positive sample was purified using AMPure XP (Angecourt), following the manufacturer’s protocol. The DNA was resuspended in 20 μL of elution buffer and stored at −20°C until sequencing by the Sanger method.

The obtained sequence was analyzed using CLC DNA Workbench 6.0 software and compared with existing sequences using the Basic Local Alignment Search Tool (BLAST). The final sequence was deposited in the GenBank database (NCBI) under the accession number: OM401930. Phylogenetic analysis was performed using *T. gondii*; *Neospora caninum* sequence data were obtained from GenBank. The sequences were aligned, and a phylogenetic tree was constructed to evaluate the relationships between the isolate identified in this study and the other sequences from different countries and animal species.

## 3. Results

**FIGURE 1:**
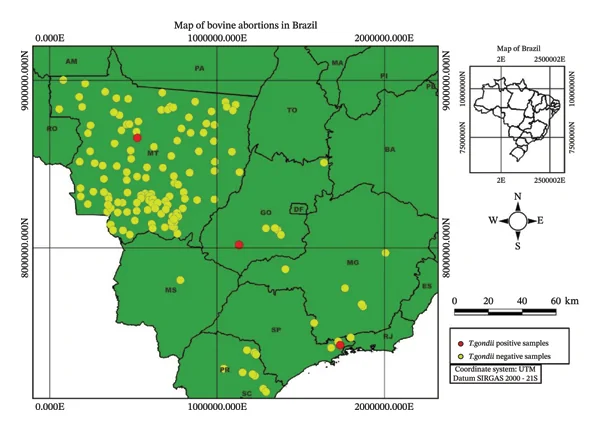
Geographic distribution of the samples.

**FIGURE 2:**
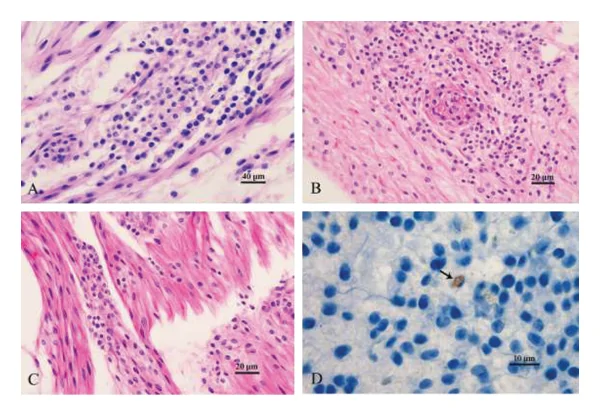
(A) Muscle: marked inflammatory cellular infiltrate, compound by mononuclear cellular infiltration, mainly lymphocytes and a few plasma cells (H&E. × 400 magnification). (B) Connective tissue: marked inflammatory cellular infiltrate, compound by lymphocytes and scattered plasma cells and macrophages (histiocytes) (H&E. × 200 magnification). (C) Muscle: marked inflammatory cellular infiltrate, compounded by macrophages (histiocytes) and lymphocytes (H&E. × 200 magnification). (D) Muscle: mononuclear infiltrate exhibiting strong immunolabeling indicative of *Toxoplasma gondii* zoites (arrow).

A total of 156 aborted bovine fetuses from different regions of Brazil were analyzed during the study period. The geographical distribution is shown in Figure 1. Histopathological examination results of samples with lesions compatible with *T. gondii* infection are available in the Supporting Information (Table S1: histopathological findings in cases of bovine abortion containing lesions compatible with *T gondii* infection [semiquantitative analysis]). Three samples (1.9%) tested positive by PCR and exhibited lesions compatible with *T. gondii.* The fetuses exhibited varying degrees of autolysis, and one fetus exhibited mummification. Clinical and epidemiological data, along with histopathological lesions, are depicted in Table 2. These samples were negative for all other pathogens tested. PCR sequencing was performed on the positive samples (Sample 1), and DNA alignment showed 100% identity with *T. gondii* DNA (GenBank MK521885.1). Tissue quantification revealed 476 copies/mg in Sample 1, 148 copies/mg in Sample 2, and 16 copies/mg in Sample 3. Immunohistochemical analysis showed immunostaining in only one of the PCR‐positive samples (Sample 3) (Figure 2). The lack of immunohistochemistry (IHC) sensitivity in the other two cases, despite positive molecular detection, may be attributed to the lower parasitic load or local distribution of the parasite in the tissue sections examined, highlighting the superior sensitivity of qPCR.

**TABLE 2:** Clinical, epidemiological, pathological, and laboratory findings in aborted fetuses due to toxoplasmosis.

Sample ID	Fetus age (months)	Location	GenBank accession	Histological lesions	Animal breed
Sample 1	5	Santa Helena de Goiás‐GO	OM401930	Mild mononuclear encephalitis with areas of mild multifocal gliosis	Holstein
Sample 2	6	Campo Verde‐MT	—	Moderate multifocal nonsuppurative encephalitis and mild multifocal monosuppurative vasculitis	Holstein
Sample 3	3	Poços de Caldas‐MG	—	Moderate multifocal mononuclear pneumonia and areas of moderate multifocal necrosis	Holstein

**FIGURE 3:**
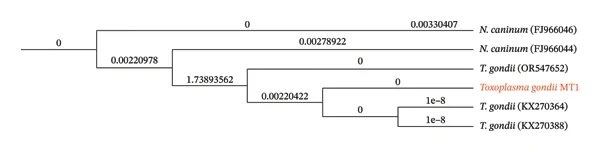
Phylogenetic tree.

Phylogenetic analysis (Figure 3) revealed that the *T. gondii* sequence obtained in this study clustered closely with other B1 gene sequences from different isolates, indicating high genetic similarity among the strains circulating in these regions.

## 4. Discussion

*T. gondii* found in fetuses was reported in the states of Goiás and Mato Grosso, in the Midwest region of Brazil, and in the state of Minas Gerais, in the Southeast region. These regions are characterized by continental tropical and high‐altitude tropical climates, respectively, which provide favorable conditions for the development of this protozoan [16].

To the best of our knowledge, this is the first documented case of bovine abortion linked to *T. gondii* in Brazil. While some studies have previously addressed this issue, most were based on serological data. Notably, Goiás showed the highest rate of positive antibody results, about 89.1%, indicating significant exposure of cattle to *Toxoplasma* in that region. It is important to note, however, that seroprevalence alone does not necessarily confirm active infection, but it does suggest widespread parasite presence on Brazilian farms [17].

The detection rate of *T. gondii* in bovine fetuses in this study (1.9%) is relatively low compared to abortion rates in small ruminants, which is consistent with previous literature indicating that cattle are more resistant to clinical toxoplasmosis. The differences in detection rates may be due to variations in management practices, environmental conditions, and the presence of definitive hosts (cats) on the farms.

The frequency of bradyzoites in cattle is significantly lower compared to pigs, sheep, and goats. Therefore, the main concern regarding *Toxoplasma* in cattle lies in maintaining the parasite on the property via reservoirs and the potential for significant economic losses due to abortions and the disposal of contaminated carcasses [18].

It is noteworthy that the affected animals were Holstein cows, which are used for milk production. On Brazilian dairy farms, it is quite common to raise cats alongside cows. Since cats are the definitive hosts of the parasite, this cohabitation significantly increases the likelihood of infection among dairy cattle [19]. In addition, other studies have detected *T. gondii* antibodies in a variety of wild animals. Of particular importance is *Hydrochoerus hydrochaeris* (capybara), which can act as a major reservoir for the protozoan. This species is present in large numbers in the region and may play a significant epidemiological role in contaminating livestock by contaminating pastures and water sources with their carcasses. [20].

In this study, the fetuses that tested positive for *T. gondii* were between 3 and 6 months old. This aligns with previous studies suggesting that *T. gondii* infection can cause abortion during various stages of pregnancy, although it is often more commonly observed in some samples (as low as 16 copies/mg), which could represent incidental presence, but the concurrent histopathological lesions strongly suggest that *T. gondii* was the primary cause of abortion in these cases.

The histopathological lesions observed in fetal tissues, as shown in Table 1 (Supporting Information), indicate areas where protozoan‐suggestive damage was identified. Such alterations are also reported in abortions caused by *Neospora caninum*, and cross‐reactions in serological testing have been documented. However, in this study, all samples were tested for other common bovine abortifacients, including *Neospora caninum*, and none were detected. This confirms that the abortions were not caused by those agents but by another agent, likely *T. gondii,* with similar histological effects [21, 22].

IHC results revealed the presence of the agent in heart tissue and damaged tissue in Sample 3. Nevertheless, it is essential to perform molecular tests, as done in this study, due to the high sensitivity of such methods compared to IHC, especially when using polyclonal antibodies, which may cross‐react with agents such as *Neospora* spp. Moreover, this underscores the need for further studies on the zoonotic potential of *Toxoplasma* and the associated risk of transmission through meat consumption, as highlighted by Silva and collaborators [23].

The phylogenetic analysis conducted in this study provides valuable insights into the genetic diversity of *T. gondii* strains causing bovine abortion. The clustering of our isolate with other known strains from the region highlights the endemic nature of these specific genetic variants and their potential role in reproductive failure in cattle.

One of the significant limitations of this study is the inability to determine the genotypes of the *T. gondii* strains causing bovine abortion. The clustering of our isolate with other known strains from the region highlights the endemic nature of these specific genetic variants and their potential role in reproductive failure in cattle.

## 5. Conclusion

Through molecular detection, identification of pathological lesions, and quantification of the agent in abortion cases, this study demonstrates that circulating *T. gondii* in Brazil—despite being primarily known through seroprevalence—is also capable of causing abortion in cattle. This highlights the need for specific prophylactic measures against the parasite, such as preventing cats from accessing animal feeding areas, not providing animal byproducts to livestock, separating or culling contaminated females, and using only previously verified semen and embryos for *in vitro* fertilization. Also, it is important to avoid management practices that facilitate its establishment. Although reported cases remain relatively few, early diagnosis can support better control of the agent.

## Funding

This study was supported by the Coordenação de Aperfeiçoamento de Pessoal de Nível Superior and the Conselho Nacional de Desenvolvimento Científico e Tecnológico (314068/2020‐1).

## Ethics Statement

The experiments were carried out in accordance with the ethics committee on the use of animals at UFMT (SEI No. 23108.024025/2023‐09).

## Conflicts of Interest

The authors declare no conflicts of interest.

## Supporting Information

Additional supporting information can be found online in the Supporting Information section.

## Supporting information


**Supporting Information** Table S1: Information about the analyzed fetuses, as well as the macroscopic conditions observed at necropsy and the lesions observed in the histopathological examination, along with a score for the degree of injury presented.

## Data Availability

The data that support the findings of this study are available from the corresponding author upon reasonable request.
