# Supplementary material for: Molecular Diagnosis and Quantification of Bovine Abortion Caused by Toxoplasma gondii in the Tropical Climate of Brazil
Source: Vet Med Int. 2026 Aug 26;2026:4914633. doi: 10.1155/vmi/4914633 (PMC13508022; doi:10.1155/vmi/4914633)
Supplement: Supplementary file 1 [file VMI-2026-4914633-s001.docx]

S1- Histopathological findings in cases of bovine abortion containing lesions compatible with *Toxoplasma gondii* infection (Semiquantitative Analysis)

| **Case** | **Year** | **Fetal age (months)** | **Macroscopic condition** | **Tissue with Lesions** | **Histopathological Lesions (Semiquantitative Scores)** |
| --- | --- | --- | --- | --- | --- |
| 1 | 2013 | 5 | Autolysed | Brain, skeletal muscle, heart and lung | Encephalitis: 1  Gliosis: 1  Myositis: 2  Myocarditis: 1  Pneumonia: 1 |
| 2 | 2013 | 6 | Autolysed | Brain, heart and lung | Encephalitis: 3  Myocarditis: 2  Pneumonia: 1 |
| 3 | 2014 | 3 | Mummification | Lung and skeletal muscle | Pneumonia: 1  Myositis: 3 |

- **"Mild"** → **Score 1**
- **"Mild to moderate"** → **Score 2**
- **"Moderate"** → **Score 3**
- **Not mentioned/Absent** → **Score 0**
